# Complete genome sequences of *Clostridium perfringens* Del1 strain isolated from chickens affected by necrotic enteritis

**DOI:** 10.1186/s13099-017-0217-6

**Published:** 2017-11-21

**Authors:** Charles Li, Xianghe Yan, Hyun S. Lillehoj

**Affiliations:** 1 Animal Biosciences and Biotechnology Laboratory, Beltsville Agricultural Research Center, Agricultural Research Service-US Department of Agriculture, Beltsville, 10300 Baltimore Avenue, MD 20705 USA; 2 Environmental Microbial and Food Safety Laboratory, Beltsville Agricultural Research Center, Agricultural Research Service-US Department of Agriculture, Beltsville, 10300 Baltimore Avenue, MD 20705 USA

**Keywords:** *Clostridium perfringens*, CP Del1 strain, Complete genome sequence

## Abstract

**Background:**

*Clostridium perfringens* is ubiquitous in nature. It is a normal inhabitant in the intestinal tract of animals and humans. As the primary etiological agent of gas gangrene, necrosis and bacteremia, *C. perfringens* causes food poisoning, necrotic enteritis (NE), and even death. Epidemiology research has indicated that the increasing incidence of NE in poultry is associated with the withdrawal of in-feed antibiotic growth promoters in poultry production in response to government regulations. The recent omics studies have indicated that bacterial virulence is typically linked to highly efficient conjugative transfer of toxins, or plasmids carrying antibiotic-resistance traits. Currently, there is limited information on understanding of host–pathogen interaction in NE caused by virulent strains of *C. perfringens*. Elucidating such pathogenesis has practical impacts on fighting infectious diseases through adopting strategies of prophylactic or therapeutic interventions. In this report, we sequenced and analyzed the genome of *C. perfringens* Del1 strain using the hybrid of PacBio and Illumina sequencing technologies.

**Results:**

Sequence analysis indicated that Del1 strain comprised a single circular chromosome with a complete 3,559,163 bp and 4 plasmids: pDel1_1 (82,596 bp), pDel1_2 (69,827 bp), pDel1_3 (49,582 bp), and pDel1_4 (49,728 bp). The genome had 3361 predicted coding DNA sequences, harbored numerous genes for pathogenesis and virulence factors, including 6 for antibiotic and antimicrobial resistance, and 3 phage-encoded genes. Phylogenetic analysis revealed that Del1 strain had similar genome and plasmid sequences to the CP4 strain.

**Conclusion:**

Complete chromosomal and plasmid sequences of Del1 strain are presented in this report. Since Del1 was isolated from a field disease outbreak, this strain is a good source to identify virulent genes that cause many damaging effects of Clostridial infections in chicken gut. Genome sequencing of the chicken pathogenic isolates from commercial farms provides valuable insights into the molecular pathogenesis of *C. perfringens* as a gastrointestinal pathogen in food animals. The detailed information on gene sequencing of this important field strain will benefit the development of novel vaccines specific for *C. perfringens*-induced NE in chickens.

**Electronic supplementary material:**

The online version of this article (10.1186/s13099-017-0217-6) contains supplementary material, which is available to authorized users.

## Background

*Clostridium perfringens* (CP) is a rod-shaped, spore-forming, Gram-positive anaerobic bacterium ubiquitous in nature and a large commensal bacterial population present in the intestines of humans and animals [1, 2]. Annual economic losses to the world poultry industry associated with CP infections are estimated to be more than $6 billion [3]. As the primary etiological agent of necrosis, bacteremia, and gas gangrene, CP causes food poisoning, necrotic enteritis (NE), chronic underperformance in commercial animal production, and even mortality [4, 5]. Isolates of CP are commonly classified into Types A to E based on the toxins produced, and virulence is mainly attributed to their individual capacities to produce multiple toxins and extracellular enzymes [1, 6]. Literature mining has been used to clearly demonstrate that horizontal gene transfer via plasmids and other extra-chromosomal elements can convert non-toxigenic CP strains into toxin producers [7, 8]. In this study, the whole-genome sequencing of CP Del1 strain and comparative genomic analyses to published sequences are presented. Ultimately, genomic sequencing of different strains will enhance our understanding of the process by which commensal bacteria such as CP evolves as an intestinal pathogen in food animals. This will lead to the definition of putative vaccine targets to better protect chickens against CP-induced NE.

## Methods

### Bacterial strain, culture conditions and DNA isolation

A virulent CP Del1 strain (BioSample accession: SAMN06252079) was originally isolated from the intestines of a chicken on an NE-affected farm in Delaware, USA in 2009 and this strain was used to induce experimental NE in broiler chickens [3]. Bacteria were cultured anaerobically for 16 h and pellets were collected for DNA extraction using the cetyl trimethylammonium bromide method [9].

### Genomic DNA sequencing

The complete genome sequence of CP Del1 was determined with the PacBio RS II platform (Pacific Biosciences, Menlo Park, CA, USA), performed and assembled at Genomic Resource Center, University of Maryland (Baltimore, MD, USA). A 20-kb DNA library was constructed using SMRTbell Template Preparation kit 1.0 according to the manufacturer’s protocol and sequenced using single-molecule real-time (SMRT) sequencing technology with P6-C4 chemistry and 1 RS II SMRT cell, and the data were assembled de novo using the hierarchical genome assembly process (HGAP). Since PacBio sequencing has a higher single-pass error rate (15%) than HiSeq platform does (1.5%) [10], the assembled whole-genome shotgun sequences were verified with Illumina HiSeq 4000 (San Diego, CA, USA) performed by Novogene Inc. (Sacramento, CA, USA). Sequencing libraries were generated using NEBNex DNA Library Prep Reagent Set (New England Biolabs Inc, Ipswich, MA, USA) and index codes were added to each sample. The clustering of the index-coded samples was performed on a cBot Cluster Generation System using HiSeq 3000/4000 PE Cluster Kit. After cluster generation, the libraries were sequenced on a HiSeq 4000 platform into paired-end 150-bp short reads.

### Assembly, genome annotation and genomic analysis

Annotation was carried out by the NCBI Prokaryotic Genome Annotation Pipeline (released 2013). Information about this annotation pipeline can be found through the following web link: https://www.ncbi.nlm.nih.gov/genome/annotation_prok.

### Quality assurance

Genomic DNA was extracted from pure cultures of a single bacterial colony of Del1. The bacterium was identified as CP by biochemical identification kit and PCR product sequencing targeting 16S rRNA and necrotic enteritis B-like toxin (netB) gene, as described previously [3]. Potential contamination of the genomic libraries by other microorganisms was evaluated using a BLAST search against the non-redundant database. A5-miseq includes a quality-checking step that detects putative mis-assemblies by identifying clusters of read pairs that map to disjointed locations in the assembled genome. This method did not detect any putative mis-assemblies. The internal sequencing errors resulting from Pacbio sequencing were corrected and verified by Illumina sequencing.

## Results and discussion

### General genome characteristics

**Fig. 1:**
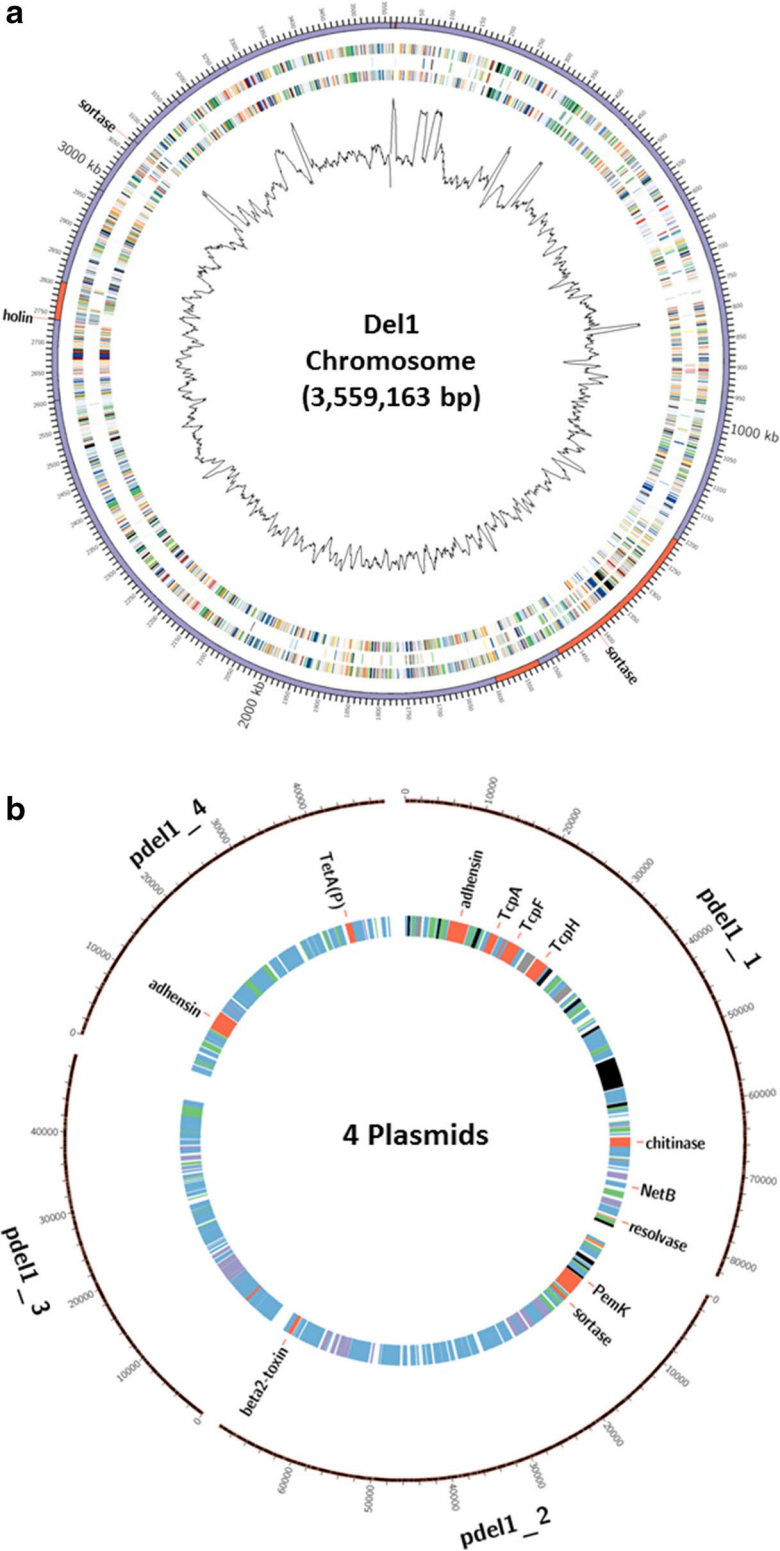
Genomics characterization of *C. perfringens* (CP) strain Del1. **a** Genome comparison between CP Del1 and reference ATCC13124 strains; **b** 4 plasmids in Strain Del1 using CIRCOS. The cut off value of BLASTN homology was 90% in Fig. 1a. Outer ring: scale, 1st ring: all genes of Del1; 2nd ring: genes less than 90% homology to ATCC13124; 3rd ring: genes more than 90% identity to ATCC13124; 4th ring: indicating the G + C content. Note: the size of Fig. 1a, b are not proportional to their actual nucleotide size

The complete genome of CP Del1 was composed of a circular chromosome (Fig. 1a) and 4 plasmids (Fig. 1b). The genome had a chromosome of 3,559,163 bp long with a GC content of 28.3%, 3556 genes (chromosome and plasmids), 3361 coding DNA sequences (CDSs) (chromosome), 30 rRNA genes (10 5S, 10 16S, and 10 23S), 94 tRNA genes and 4 non-coding RNAs (Table 1). The plasmids, designated as pDel1_p1, pDel1_p2, pDel1_p3 and pDel1_p4, were 82,596, 69,827, 49,582 and 49,728 bp long, respectively. The genomic annotation data for CP Del1 and 3 other CP reference strains, CP4, JP55 and ATCC13124, are summarized in Table 1. One striking difference was that CP4 had only 38 RNA genes while the 3 other CP strains contained 128 genes. This discrepancy may be accounted for by incomplete gene sequences of CP4 strain.

**Table 1:** Summary in genome annotation data for *Clostridium perfringens* strain Del1 and 3 reference strains: CP4, JP55 and ATCC13124

Genome annotation	Clostridium perfringens Strains			
	DEL1	CP4	JP55	ATCC13124
Genome accession#	NZ_CP019576.1 (3559163 bp)	GCA_001414595.1 (latest)	NZ_CP010993.1 (3347300 bp)	NC_008261.1 (325666 bp)
Sequence status	Complete genome	Contigs (98 contigs)	Complete genome	Complete genome
Annotation date	3/5/2017	04/04/2017	04/05/2017	04/06/2017
Assembly method	Celera Assembler & Genomics Workbench v. V. 9.5.1	Velvet v. 0.7.48	DNASTAR SeqMan v. NGEN12	na
Annotation method	Best-placed reference protein set; GeneMarks+	Best-placed reference protein set; GeneMarks+	Best-placed reference protein set; GeneMarks+	Best-placed reference protein set; GeneMarks+
Annotation software revision	4.1	4.1	4.1	4.1
Genes (total)	3556	3394	3287	2948
CDS (chromosome)	3361	3293	3064	2801
Genes (RNA)	128	38	128	128
rRNAs	10, 10, 10 (5S, 16S, 23S)	1 (5S)	10, 10, 10 (5S, 16S, 23S)	8, 8, 8 (5S, 16S, 23S)
tRNAs	94	33	94	27
ncRNAs	4	4	4	4
Pseudo genes (total)	136	106	95	27

The Clusters of Orthologous Group (COG)-associated functional genes for CP Del1 and other bacterial starins are shown in Additional file 1: Table S1. Among the various COG categories in CP Del1, 6 comprised the largest proportions (each ≥ 5% of the total COG classifications) in order from largest to least: R [general function prediction only, 319 open reading frame (ORFs), 9.76%], S (function unknown, 283 ORFs, 8.65%), G (carbohydrate transport and metabolism, 203 ORFs, 6.21%), K (transcription, 202 ORFs, 6.18%), E (amino acid transport and metabolism, 181 ORFs, 5.54%), and J (translation, ribosomal structure and biogenesis, 169 ORFs, 5.17%). Generally, all CP strains (Del1, JP55) exhibited comparable ranges in proportions of COG classifications.

### Comparative phylogenetic analysis and genomic variants among *C. perfringens* strains

**Fig. 2:**
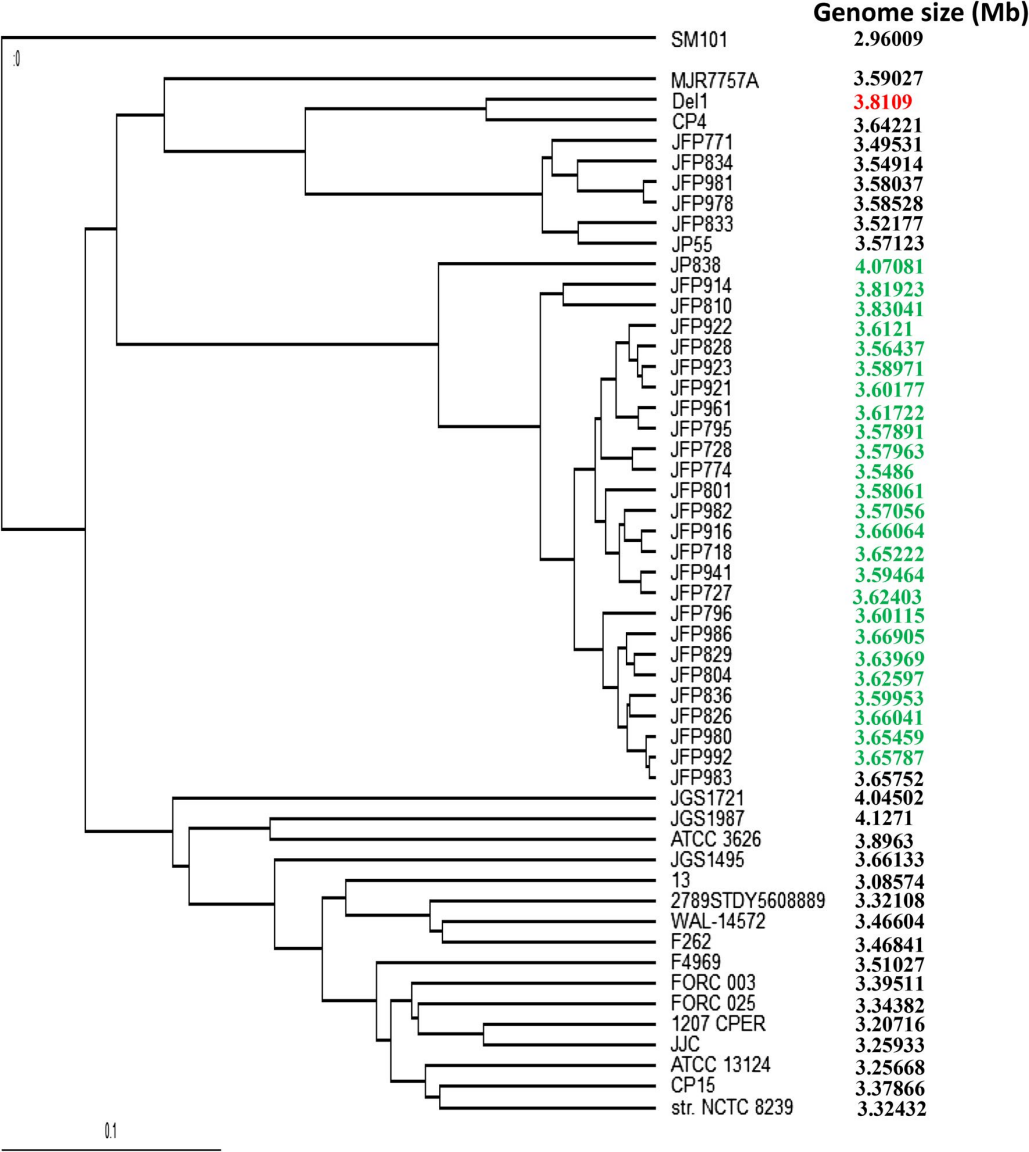
Phylogenetic tree analysis of CP Del1 and other 51 published *C. perfringens* reference strains in genomic dendrogram by orthologous average nucleotide identity (ANI). The genome sizes are listed in the right

To date, 52 complete genome sequences of CP strains and their annotation data are available in the GenBank database. To compare these genomes, whole genome DNA-sequence-based average nucleotide index (ANI) analysis was performed and phylogenetic trees were drawn based on genomic sequence analysis of these 52 CP genomes (Fig. 2). The dendrogram illustrates that CP Del1 strain was the most closely related to CP4 strain, and clustered with JP55, MJR7757A and 5 other JFP strains. CP4 strain, whose genomic sequence has been characterized (9), is a pathogen that causes NE [11], and contains 3 highly conserved NE-associated loci that are designated NELoc-1 (42 kb), NELoc-2 (11.2 kb) and NELoc-3 (5.6 kb), with netB residing on an ~ 42-kb plasmid-encoded pathogenicity locus (NELoc-1) [12]. In CP Del1, netB gene resides in plasmid pDel1_1.

### Pathogenesis and virulence factors

We also found numerous important genes involved in pathogenesis and virulence in the CP Del1 chromosome and plasmids (Additional file 1: Table S2). CP Del1 and CP4 strains share similar genes, with more genes absent in the strain ATCC13124 genome. Besides alpha-toxin phospholipase C (cpa plc), CP Del1 genome also had genes encoding necrotic enteritis B-like toxin (netB), Beta 2 toxin (cpb2), mu-toxin (nagH-K), sialidase (nanH, nanI and nanJ), collagenase (peptidase_U32), and perfringolysin O (thera toxin, pfo). Alpha toxin (cpa-encoded) is the most toxic extracellular enzyme produced by CP type A. It is present in all types and essential for virulence, and its core protein, phospholipase C, hydrolyzes both important constituents of eukaryotic cell membranes: phosphatidylcholine and sphingomyelin [13, 14]. NetB is a pore-forming toxin critical for induction of NE in chickens with predisposing factors, such as preceding coccidial infection and dietary manipulation [15, 16]. Sialidase cleaves terminal sialic acid residues in the alpha configuration linked to oligosaccharide chains present on proteins and lipids and functions as a nutritional factor to support bacterial growth and as a virulence factor during bacterial pathogenesis [1].

Studies on the genetic diversity of *C. perfringens* in mild NE-affected chickens indicated that more than 90% of all isolates from NE-specific organ lesions carried netB gene [17]. However, immunization with vaccines containing recombinant NetB toxin only partially protected progeny chickens from necrotic enteritis challenges [18]. Other virulence factors may additively contribute to the CP pathogenesis in NE induction. Global transcriptomic analysis performed on ligated intestinal loops in chickens following infection with a netB^+^ CP strain revealed that numerous virulence factors were significantly expressed in vivo, such as cpa, netB and sialidase genes [19]. Del1 strain is a virulent strain isolated from a field disease outbreak to be used as a challenge strain. Compared to CP4 strain, this Del1 strain contains some unique genes expressing adhensin, pilin of the toxin- coregulated-pilus (tcp cluster C–J), and programmed cell death toxin (PemK) which may be involved in multitude of pathogenesis functions, including adherence to eukaryotic cells, DNA uptake, and protein/toxin secretion, and therefore enhancing the pathogenesis and favoring the colonization and infection of *C. perfringens* as a gastrointestinal pathogen in food animals. Sequencing analysis also indicated that Del1 strain did not contain genes expressing Tpel toxin and surface protein CPE1231 as the CP4 strain had. Previously, it was reported that Tpel protein enhanced bacterial virulence and severity of NE [16]. Identifying bacterial important genes that encode toxin and virulence factors in genomics and transcriptomics approaches is critical for recombinant vaccine development, for example, cpa, netB, sialidase B, tpel, and PemK. In general, these genes contribute to toxin generation and bacterial colonization and growth. Strain Del1 is classified as Type A due to absence of beta, iota and epsilon toxins based on the definition of the presence of toxin genes in toxinotyping [1].

### Antibiotic-resistance genes

By screening the genome and plasmid sequences against the GenBank library, we identified 6 antibiotic-resistance genes/clusters in CP Del1 genomes and plasmids. Specially these genes conferred resistance to tetracycline (total 3 genes: 1 gene encoding tetracycline ribosomal protection with Accession No: YP_001967743, 2 genes encoding tetracycline efflux pumps: BAB71966 and YP_001966009), 1 gene for class B beta-lactams (Subclass B1_136), as well as 1 gene for other antibiotics (YP_698526). Finally, another 1 gene encoded a major facilitator superfamily that was involved with antibiotic efflux pump (P96712), by which membrane transport proteins facilitated movement of small antibiotics across the cell membrane [20]. CP4 was also found to confer resistant to tetracycline [21]. Del1 strain was found in its susceptibility test to be resistant to tetracycline hydrochloride with minimal inhibitory concentrations of more than 256 µg/mL.

### Identification of phage-encoded genes in Del1 strain

Bacteriophage DNAs serve as mobile genetic elements which confer the antibiotic-resistance genes or virulence determinants by transduction, and ultimately contribute to genome diversification of bacterial hosts, with strain to strain variations in bacteriophage repertoire [22]. However, host specificity has routine been observed relative to the bacteriophages isolated from various CP strains. This is most likely due to evolution of the receptor in the bacterial hosts and anti-receptor molecules in bacteriophages (3). Using a previously described bioinformatics platform [23], we have determined specific genes in two regions (1224604–1235574 and 2737554–2740876) in Del1 genome which have high sequence similarities to *Clostridium* phage *vB_CpeS*-*CP51*, *Clostridium* phage *PhiS63*, and bacteriophage *phi3626*. Further, these regions also have low homology between them, indicating the prophages in Del1 strain are also strain specific. Sequence annotation predicts that majority of the genes in these two prophages-like areas encode hypothetical proteins.

## Future directions

We have obtained a complete genome and 4 associated plasmid sequences of CP Del1 strain. The genomic analysis revealed that CP Del1 sequences consist of numerous genes encoding toxins and virulence factors, and include 6 that encode for proteins to confer antibiotic resistance, and 3 bacteriophage gene inserts. The improved understanding of the molecular pathogenesis of CP-induced NE in chickens is necessary for development of effective treatments. Future studies should identify specific roles of pathogenesis/virulence factors, especially those present in the plasmids. Ultimately, we envision that this data and approach will guide us to the selection of strains and CP gene targets for further vaccine development.

## Additional file

**Additional file 1: Table S1.** Numbers and proportions of general COG-associated functional genes for *Clostridium perfringens* strains Del1 and JP55. **Table S2.** The comparisons of virulence genes among *Clostridium perfringens* CP Del1, and 2 reference strains CP4 and ATCC13124.

## Abbreviations

CP: *Clostridium perfringens*
NE: necrotic enteritis
netB: necrotic enteritis toxin B-like
CDS: coding DNA sequence
COG: clusters of orthologous group
ANI: average nucleotide identity
