# Supplementary material for: Complete genome sequences of Clostridium perfringens Del1 strain isolated from chickens affected by necrotic enteritis
Source: Gut Pathog. 2017 Nov 21;9:69. doi: 10.1186/s13099-017-0217-6 (PMC5699181; doi:10.1186/s13099-017-0217-6)
Supplement: Supplementary file 1 — Additional file 1: Table S1. Numbers and proportions of general COG-associated functional genes for Clostridium perfringens strains Del1 and JP55. Table S2. The comparisons of virulence genes among Clostridium perfringens CP Del1, and 2 reference strains CP4 and ATCC13124. [file 13099_2017_217_MOESM1_ESM.docx]

Additional Files

Table S1. Numbers and proportions of general COG-associated functional genes for *Clostridium perfringens* strains Del1 and JP55

| **Code** | **Description** | **Del1** | | **JP55** | |
| --- | --- | --- | --- | --- | --- |
|  |  | **Value** | **% of total** | **Value** | **% of total** |
| **A** | RNA processing and modification | 1 | 0.03 | 1 | 0.03 |
| **B** | Chromatin structure and dynamics | 1 | 0.03 | 1 | 0.03 |
| **C** | Energy production and conversion | 128 | 3.91 | 129 | 4.23 |
| **D** | Cell cycle control, cell division, chromosome partitioning | 55 | 1.68 | 53 | 1.74 |
| **E** | Amino acid transport and metabolism | 181 | 5.54 | 179 | 5.87 |
| **F** | Nucleotide transport and metabolism | 89 | 2.72 | 90 | 2.95 |
| **G** | Carbohydrate transport and metabolism | 203 | 6.21 | 216 | 7.08 |
| **H** | Coenzyme transport and metabolism | 103 | 3.15 | 104 | 3.41 |
| **I** | Lipid transport and metabolism | 63 | 1.93 | 59 | 1.94 |
| **J** | Translation, ribosomal structure and biogenesis | 169 | 5.17 | 161 | 5.28 |
| **K** | Transcription | 202 | 6.18 | 192 | 6.30 |
| **L** | Replication, recombination and repair | 154 | 4.71 | 146 | 4.79 |
| **M** | Cell wall/membrane/envelope biogenesis | 160 | 4.89 | 157 | 5.15 |
| **N** | Cell motility | 20 | 0.61 | 20 | 0.66 |
| **O** | Posttranslational modification, protein turnover, chaperones | 76 | 2.32 | 72 | 2.36 |
| **P** | Inorganic ion transport and metabolism | 117 | 3.58 | 115 | 3.77 |
| **Q** | Secondary metabolites biosynthesis, transport and catabolism | 26 | 0.80 | 23 | 0.75 |
| **R** | General function prediction only | 319 | 9.76 | 297 | 9.74 |
| **S** | Function unknown | 283 | 8.65 | 259 | 8.49 |
| **T** | Signal transduction mechanisms | 121 | 3.70 | 120 | 3.94 |
| **U** | Intracellular trafficking, secretion, and vesicular transport | 47 | 1.44 | 50 | 1.64 |
| **V** | Defense mechanisms | 60 | 1.83 | 59 | 1.94 |
| **W** | Extracellular structures | 0 | 0.00 | 0 | 0.00 |
| **Y** | Nuclear structure | 0 | 0.00 | 0 | 0.00 |
| **Z** | Cytoskeleton | 1 | 0.03 | 1 | 0.03 |
| - | not in COGs | 691 | 21.13 | 545 | 17.87 |
| Total protein |  | 3270 |  | 3049 |  |
|  |  |  |  |  |  |

Shaded fields indicate the proportion of more than 5% of total COG-associated functional genes.

Table S2. The comparisons of virulence genes among *Clostridium perfringens* CP Del1, and 2 reference strains CP4 and ATCC13124.

| **Gene_Symbol** | **Toxinotypes/biomarkers** | **Del1** | **CP4** | **ATCC13124** |
| --- | --- | --- | --- | --- |
| adhensin | Adhensin | yes | no | no |
| cna | Collagen adhensin | yes | yes | no |
| colA | Collagenase (κ-toxin), | yes | yes | yes |
| cpa (Plc) | Alpha-toxin (A, B, C, D, E) | yes | yes | yes |
| cpb | Beta-toxin (CPB) | no | no | no |
| cpb2 | Beta2-toxin | yes | yes | no |
| cpe | Enterotoxin (A, C, D, E) | no | no | no |
| delta-toxin | Delta-toxin | no | no | no |
| etx | Epsilon-toxin (ETX) | no | no | no |
| cpe0378 | Myosin-crossreactive antigen | yes | yes | yes |
| fbp | Probable Fibronectin-binding protein | yes | yes | yes |
| cloSI | Alpha-clostripain | yes | yes | yes |
| cpe1231 | Surface protein | no | yes | no |
| cpe1847 | Fibronectin-binding protein | yes | yes | yes |
| cpe2158 | Probable adhensin | yes | yes | yes |
| cpe2281 | Type IV pili | yes | yes | yes |
| pilC | Type IV pili | yes | yes | yes |
| tapB | Type IV pili | yes | yes | yes |
| entA | Enterotoxin | yes | yes | yes |
| entB | Enterotoxin | yes | yes | yes |
| entC | Enterotoxin | no | no | no |
| entD | Enterotoxin | yes | yes | yes |
| groEL | GroEL (extracellular protein) | yes | yes | yes |
| hlyA | Hemolysin-related protein | yes | yes | yes |
| hlyB | Hemolysin | yes | yes | yes |
| hlyC | Hemolysin | yes | yes | yes |
| hlyD | Hemolysin | yes | yes | yes |
| hlyE | Hemolysin III | yes | yes | yes |
| iap | Iota-toxin (ITX) | no | no | no |
| ibp | Iota-toxin (ITX) | no | no | no |
| nagH | Mu-toxin | yes | yes | yes |
| nagI | Mu-toxin | yes | yes | yes |
| nagJ | Mu-toxin | yes | yes | yes |
| nagK | Mu-toxin | yes | yes | yes |
| nagL | Mu-toxin | yes | yes | no |
| nanH | Sialidase | yes | yes | yes |
| nanI | Sialidase | yes | yes | yes |
| nanJ | Sialidase | yes | yes | yes |
| netB | Hemolytic, Necrotizing enteritis in chickens | yes | yes | no |
| pemK | Programmed cell death toxin YdcE | yes | no | no |
| peptidase_U32_C | Collagenase | yes | yes | yes |
| peptidase_U32 | Collagenase | yes | yes | yes |
| pfo(pfoA) | A, B, C, D, E | yes | yes | yes |
| putative bacteriocin | Bacteriocin (WP_003470134.1) | no | no | no |
| tcpA | DNA translocase or  coupling protein | yes | yes | no |
| tcp cluster (C, D, E, F, G, H, I, J) | Major pilin of the toxin- coregulated-pilus | yes | no | no |
| tetA(P) | Antibiotics resistance | yes | yes | no |
| tetB(P) | Antibiotics resistance | yes | yes | no |
| tpeL | Cytotoxin | no | yes | no |
| virR | VirR | yes | yes | no |
| virS | VirS | yes | yes | no |
